# Neuroprotective Effects of Calpain Inhibition in Parkinson’s Disease: Insights from Cellular and Murine Models

**DOI:** 10.3390/cells14171310

**Published:** 2025-08-24

**Authors:** Vandana Zaman, Amy Gathings, Kelsey P. Drasites, Donald C. Shields, Narendra L. Banik, Azizul Haque

**Affiliations:** 1 Ralph H. Johnson Veterans Administration Medical Center, 109 Bee St, Charleston, SC 29401, USA; baniknl@musc.edu; 2 Department of Neurosurgery, Medical University of South Carolina, 96 Jonathan Lucas Street, Charleston, SC 29425, USA; kelseydrasites@mail.rossmed.edu (K.P.D.); donshields@sbcglobal.net (D.C.S.); 3 Department of Pharmacology and Immunology, Medical University of South Carolina, 173 Ashley Avenue, Charleston, SC 29425, USA; amy.j.gathings@gmail.com

**Keywords:** calpain, chemokines, cytokines, MPTP, neurodegeneration, neuroinflammation Parkinson’s disease, reactive oxygen species

## Abstract

Parkinson’s disease (PD) is characterized by the progressive loss of dopaminergic neurons in the substantia nigra, and key pathways such as neuroinflammation, oxidative stress, and autophagy are believed to significantly contribute to the mechanisms of neurodegeneration. Calpain activation plays a critical role in neuroinflammation and neurodegeneration, as demonstrated by its impact on microglial activation, reactive oxygen species (ROS) production, and neuronal survival. In this study, we investigated the effects of calpain inhibition using calpeptin (CP) and calpain-2-specific inhibitors in cellular and murine models of neuroinflammation and PD. In BV2 microglial cells, LPS-induced production of pro-inflammatory cytokines (TNF-α, IL-6) and chemokines (MCP-1, IP-10) were significantly reduced by CP treatment with a concomitant decrease in ROS generation. Similarly, in VSC-4.1 motoneuron cells, calpain inhibition attenuated IFN-γ-induced ROS production and improved cell viability, demonstrating its neuroprotective effects. Moreover, in a murine MPTP model of PD, calpain inhibition reduced astrogliosis, ROCK2 expression, and levels of inflammatory cytokines (TNF-α, IL-1β, IL-6, IL-7, and IL12p70) and chemokines (MCP-1 and IP-10) in the dorsal striatum and plasma. The specific role of calpain-2 in immune modulation was further highlighted in human microglia, SV-40 cells. With respect to immune modulation in these cells, siRNA-mediated knockdown of *calpain-2*, but not *calpain-1*, significantly reduced antigen presentation to CD4+ T cells. Thus, calpain-2 is likely involved in regulating antigen presentation and activation of inflammatory CD4+ T cells. These findings underscore the therapeutic potential of calpain-2 inhibition in mitigating neuroinflammation and neurodegeneration, particularly in PD, by targeting microglial activation, ROS production, and neuronal survival pathways.

## 1. Introduction

Neurodegenerative diseases—such as Parkinson’s disease (PD), Alzheimer’s disease (AD), and amyotrophic lateral sclerosis (ALS)—are characterized by the progressive loss of neuronal structure and function, leading to debilitating motor and cognitive impairments [1,2,3]. These disorders share common pathological features, including protein aggregation, mitochondrial dysfunction, oxidative stress, and chronic neuroinflammation [4,5,6,7]. Among the molecular mechanisms driving neurodegeneration, the dysregulation of calcium homeostasis and the subsequent activation of calcium-dependent proteases, particularly calpains, appears to be critical contributors to neuronal injury and death [8,9]. Calpains are a family of non-lysosomal cysteine proteases that require calcium for activation. They are involved in a wide range of cellular processes, including cytoskeletal remodeling, signal transduction, cell cycle regulation, and apoptosis. In the nervous system, calpains play essential roles in synaptic plasticity, axonal growth, and neuronal survival under physiological conditions [10,11,12,13]. However, under pathological conditions—such as excitotoxicity, oxidative stress, or neuroinflammation—excessive calpain activation leads to the aberrant cleavage of substrates critical for neuronal integrity. For example, calpains cleave structural proteins like spectrin and tau, disrupt mitochondrial function by targeting proteins such as Bax and Bcl-2, and promote apoptosis through the activation of caspases [14,15]. These events collectively contribute to synaptic dysfunction, axonal degeneration, and neuronal death; all of these are hallmarks of neurodegenerative diseases.

The calpain family consists of 15 known isoforms, with calpain-1 (μ-calpain) and calpain-2 (m-calpain) being the most extensively studied in the context of neurodegeneration. Calpain-1 is typically activated at lower calcium concentrations and has been associated with neuroprotective functions, such as synaptic plasticity and long-term potentiation (LTP). In contrast, calpain-2 requires higher calcium concentrations for activation, and is often linked to neurotoxic processes, including excitotoxicity, inflammation, and cell death [16,17]. Calpain-2 also plays a role in tumor cell growth via the PI3K-Akt-FoxO-p27(Kip1) signaling pathway, which is a key regulator of cell proliferation [18]. The knockout of calpain-2 in mice is lethal, indicating its essential role in embryonic development [19]. Studies have shown that a calpain-2 inhibition reduces inflammation by blocking pro-inflammatory signaling pathways [20,21]. In microglia, the resident immune cells of the central nervous system (CNS), calpain activation has been implicated in the amplification of neuroinflammatory responses [22].

Our previous study of neurodegeneration in rats treated with rotenone, an animal model of PD, demonstrated increased expression of calpain-2 in dopaminergic neurons of the substantia nigra (SN) [8,23]. Treatment of these rats with a calpain inhibitor, calpeptin, reduced rotenone-induced overexpression of calpain-2 and prevented degeneration of these neurons. Moreover, the expression of calpain-1 was not significantly influenced by calpeptin treatment [8]. This dichotomy underscores the significant role of isoform-specific regulation in developing targeted therapies for neurodegenerative diseases. However, several questions remain regarding the role of calpains in neurodegeneration. For instance, the relative contributions of calpain-1 and calpain-2 to neuroinflammation and neuronal injury are not fully understood. Moreover, the therapeutic potential of isoform-specific calpain inhibition in mitigating neurodegeneration has yet to be fully explored. Addressing these questions is critical for developing effective treatments for neurodegenerative diseases, which currently lack disease-modifying therapies.

In this study, we used a variety of in vitro and in vivo models to investigate the role of calpain activation in neuroinflammation and neurodegeneration. Our findings suggest that specific activation of calpain-2 in microglia may enhance antigen presentation and T cell activation, further perpetuating neuroinflammation. These findings suggest that calpain-2 inhibition could attenuate microglial activation or microglia-triggered immune activation and its detrimental effects on neuronal survival. Our findings also demonstrate that calpain inhibition attenuates neuroinflammation, and that the calpain-2 isoform plays a critical role in modulating antigen presentation by microglia. These results not only advance our understanding of the molecular mechanisms underlying neurodegeneration but also highlight the therapeutic potential of isoform-specific calpain inhibitors in reducing neuroinflammation and neuronal damage. By targeting specific calpain isoforms, it may be possible to develop more precise and effective treatments for neurodegenerative diseases.

## 2. Materials and Methods

### 2.1. Experimental Design

**Figure 1:**
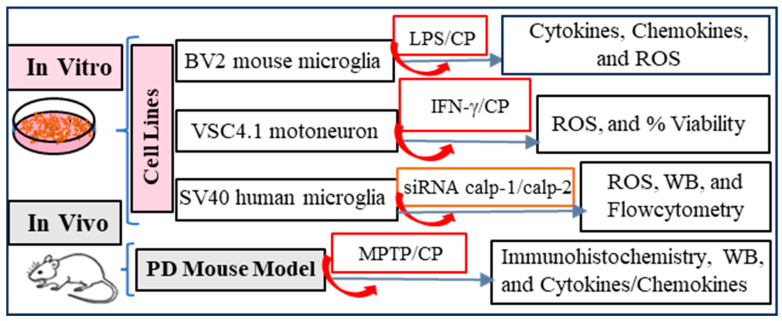
Experimental design of our study.

Our goal was to examine the role of calpain activation in neuroinflammation and neurodegeneration. To achieve this goal, we employed the experimental design shown in Figure 1. Calpain activity was blocked by treatment with calpeptin (CP), a cell-permeable inhibitor that offers several advantages such as (1) high selectivity for calpains, (2) cell permeability, (3) it is a reversible, competitive inhibitor, (4) it is stable and water soluble, and (5) it exhibits lower toxicity than other calpain inhibitors. However, there are limitations to the use of calpeptin, mainly that it is not absolutely specific, weakly inhibiting cathepsins at high concentrations, and that it has a short half-life in vivo. In other experiments, siRNA was used to selectively knock down either *calpain-1* or *calpain-2* isoforms.

A key aspect of our approach was the use of three different cell lines for in vitro experiments. These cell lines were used in various assays to evaluate the effects of pan calpain inhibition or inhibition of two major calpain isoforms (calpain-1 or calpain-2) on inflammatory parameters. Importantly, we examined the role of calpain-2 in antigen presentation by activated microglia. The MPTP mouse model used in this study is a widely used research tool for studying the pathways associated with Parkinson’s disease (PD). We used this model to evaluate the effects of a calpain inhibitor on MPTP-induced neuroinflammation in vivo.

### 2.2. Cell Lines

Murine BV2 microglial cells were grown in Isocove’s DMEM medium with L-glutamine, 25 mM HEPES without alpha-thioglycerol and beta-mercaptoethanol (Corning Inc., Corning, NY, USA cat#10-016-CV) with 10% bovine growth serum (Hyclone, Logan, UT, USA, cat #SH30541.03) and 1% penicillin & streptomycin [24]. BV2 cells were plated in 96-well black plate with clear bottom (Corning Inc., Corning, NY, USA, cat#3603) at a density 5 × 10^4^ cells/100 μL, and incubated at 37 °C with 5% CO_2_. The cells were incubated overnight with: (i) No Treatment, (ii) Lipopolysaccharide (LPS) (1 µg/mL, final concentration), (iii) Calpeptin (CP, 10 μM), and (iv) LPS+CP. All treatments were performed in triplicate. The following day, ROS assays were performed as described below.

The spinal motor neuronal hybrid cell line, VSC4.1 was also used to investigate the role of calpain inhibitor in cell survival. VSC4.1 cells were grown in DMEM/F12 50/50 medium (Corning, cat #10-092CV) with SATO’s 50XS solution as described previously [25] along with 1% penicillin-streptomycin and 2% defined bovine calf serum (Hyclone, Logan, UT, USA, cat# SH30087.03). The flasks were coated with poly-L-ornithine (Sigma, St. Louis, MO, USA, cat# A004 C). Cells were grown in the incubator at 37 °C in the presence of 5% CO_2_ in a humid chamber.

The human microglial cells SV40 were grown on collagen I, rat tail (0.1 mg/mL, Invitrogen, ThermoFisher Scientific, Waltham, MA USA, cat# A1048301) coated T-25 flasks using DMEM/F12 media (GIBCO, ThermoFisher Scientific, Waltham, MA USA, cat# 11320033) + 10% Fetal bovine serum (NEUROMICS, Edina, MN, USA, cat# FBS007) + 1% penicillin-streptomycin. The human microglia cell line SV40 transduced with HLA-DR4 was used in the antigen presentation assay. Briefly, SV40 cells were transduced using retroviral vectors for constitutive expression of HLA-DR4 (DRB1*0401) with linked drug selection markers for hygromycin and histidinol resistance [26]. The expression of surface HLA-DR4 complexes on SV40 cells was confirmed by flow cytometric analysis using the DR4-specific mAb, 359-F10 [27].

### 2.3. MTS Assay for Measuring Cell Viability

VSC4.1 cells were prepared for MTS assay and collected at 5 × 10^5^ cells/mL; 100 µL of cells were seeded into a 96-well plate [28]. Inflammatory response was induced in these cells by treating with IFN-γ (40 ng/mL). Treatments were added in triplicate: (i) Control, (ii) IFN-γ, (iii) Calpeptin (CP, 10 µM), (iv) IFN-γ+CP. Cells were incubated in them for 24 h at 37 °C. The cells in the control group received vehicle (0.01% DMSO). After 24 h, CellTiter 96 Aqueous One Solution (PROMEGA, Madison, WI, USA, cat#G3580) was used. MTS assay reagent (20 µL) was added into each well of the 96-well assay plate containing the cells with treatments in 100 µL of culture medium. The 96-well plates were then incubated for 30 min at 37° C, followed by the first absorbance reading at 490 nm using the 96-well plate reader (BioTek EL800, Agilent, Santa Clara, CA, USA). The plate was then placed back and incubated for 30 more minutes, followed by the second absorbance reading at 490 nm. Data are representative of at least three separate experiments and are expressed as cell viability ± S.D. of triplicate wells.

### 2.4. Reactive Oxygen Species (ROS) Assay

VSC4.1 cell lines were tested for ROS production following treatment with IFN-γ and calpain inhibition by calpeptin. Cells were cultured in DMEM/F12 complete media in tissue culture T75 flasks until 80% confluency. VSC4.1 cells were then collected, counted, and 5 × 10^4^ cells were plated in 96-well assay black clear bottom plate (Corning COSTAR, San Diego, CA, USA, cat# 3603). Cells were treated with IFN-γ (800 unit/mL) alone or in combination with CP (25 µM) for 24 h in 96-well plates (200 µL/well). ROS were detected using the Reactive Oxygen Species Assay Kit (Abcam, Cambridge, MA, USA, cat# ab186029) following the manufacturer’s protocol. Data analysis was performed using a two-tailed *t*-test.

### 2.5. Knockdown of Calpain-1 and Calpain-2 in SV40 Human Microglia Cells

Commercially available Santa Cruz reagents (Calpain-1 siRNA cat# sc-29885, and Calpain-2 siRNA, cat# sc-41459, Santa Cruz Biotechnology, San Diego, CA, USA) were used to knock down *calpain-1* and *calpain-2.* For siRNA induction, siRNA mix or control siRNA was made in Opti-MEM solution (2 µM). The Oligofectamine mix was made using the Opti-MEM solution as per the manufacturer’s instructions. The two mixtures sat at room temperature for 15 min. The Oligofectamine mix was added to the siRNA mix. The mix sat at room temperature for 15 min. While the siRNA+ Oligofectamine mix sat at room temperature for 15 min, SV40 cell culture media was replaced with RPMI (Mediatech Inc., Herndon, VA, USA) without serum. siRNA + Oligofectamine + Opti-MEM-RPMI mix was added to the culture and incubated at 37 °C overnight. Complete RPMI was added to the culture and the cells were incubated overnight at 37 °C for siRNA knockdown. After incubation, cells were collected to test calpain-1 and calpain-2 expression levels by Western blot, and for use in the antigen presentation assay.

### 2.6. Flow Cytometry

SV40 human microglia cells treated with scrambled siRNA, calpain-1 siRNA, or calpain-2 siRNA were washed with PBS and staining buffer (PBS+1% heat-inactivated BGS) (HyClone) and resuspended in a binding buffer (Cat# 556454, BD Biosciences: 0.1 M HEPES (pH 7.4), 1.4 M NaCl_2_, and 25 mM CaCl_2_, Franklin Lakes, NJ, USA). Cells were stained with the HLA-DR4-specific mAb, 359-F10, followed by appropriate secondary antibody labeled with the FITC as described [29]. Background fluorescence was evaluated using an irrelevant isotype-matched mAb IN-1 as described [30]. 

### 2.7. Western Blot Analysis

Cells were washed with Hank’s buffered salt solution (HBSS, Cellgro, Corning, NY, USA), and cell lysates were prepared using a laboratory standardized lysis buffer (10 mM Tris, pH 7.5, 150 mM NaCl, and 1% Triton X-100) plus protease inhibitors, phenylmethylsulfonyl fluoride (PMSF) and tosyl-L-lysine chloromethyl ketone (TLCK). Protein concentration was determined in the cell lysate using the colorimetric assay based on the Lowry assay using Bio-Rad Protein Assay Kit (DCTM Protein Assay Reagents, Hercules, CA, USA). For Western blot, 20 µg of proteins were loaded and electrophoresed on a 4–12% Bis/Tris NuPage gel (Invitrogen, Grand Island, NY, USA). The separated proteins were transferred onto a nitrocellulose membrane (Pierce, Rockford, IL, USA). The blot was probed with calpain-1 (1:500, Cell Signaling) and calpain-2 (1:500, Cell Signaling Technologies, Danvers, MA, USA, cat# 2539), NLRP3 (1:1000, Abcam, cat# ab263899), IL-6 (1:500, Santa Cruz Biotechnology, Dallas, TX, USA, cat# sc-57315), and IL-1 β (1:500, Abcam, cat#ab283818) antibodies. The monoclonal antibody for β-actin (1:500, Santa Cruz Biotechnology), or GAPDH (1:500, Santa CruzBiotechnology, cat# sc-47724) were used as a protein loading control. The secondary antibodies consisting of horseradish peroxidase-conjugated anti-mouse (1:4000, R&D Systems, Inc., Minneapolis, MN, USA, cat# HAF007) and anti-rabbit (1:2000, Santa Cruz, SC-2004) were used. Blots were incubated with SuperSignal West Femto Maximum sensitivity substrate (ThermoFisher Scientific, Rockford, IL, USA, cat# 34095) and blot images were taken using Azure Biosystems c600 Imager (Dublin, CA, USA).

### 2.8. Animals

Young adult male C57/BL6 mice (25–30 gm body weight, Charles River, Wilmington, NC, USA) were housed in the animal facility under standard conditions (12 h light-dark cycles, 23 °C, and 55% relative humidity). The mice had ad libitum access to food and water. The animals were handled and cared for in compliance with the guidelines of the National Institutes of Health (NIH, Bethesda, MD, USA) *Guide for the Care and Use of Laboratory Animals* (NIH publication 80–23, revised 1996). This study protocol was reviewed and approved (ACORP 700) by the Institutional Animal Care and Use Committee (IACUC), Ralph H. Johnson VA Medical Center, Charleston, SC, USA.

### 2.9. MPTP Mouse Model

Mice were selected randomly for MPTP (SIGMA-Aldrich, St. Louis, MO, USA) sub-chronic treatment. Animals were divided into three different treatment groups: (i) Vehicle Control, (ii) MPTP, and (iii) MPTP+CP. MPTP was dissolved in normal saline and administered intraperitoneally (i.p.) at a dose of 25 mg/kg body weight for 5 days. Calpeptin (CP, SIGMA-Aldrich, St. Louis, MO, USA), a pan calpain inhibitor, was dissolved in dimethyl sulfoxide (DMSO, SIGMA-Aldrich) to make a stock solution. The stock solution was diluted in saline to make a working solution, and mice in the MPTP+CP treatment group received calpeptin at a dose of 25 µg/kg body weight. Disposable cages and supplies were used to house MPTP-treated mice during the treatment. Mice were transferred to regular cages after 72 h post-MPTP treatment.

### 2.10. Animal Tissue Sample Collection

Animals were sacrificed at 8–10 days post-MPTP treatment. Blood samples were collected in BD Vacutainer EDTA-coated tubes for plasma (BD Vacutainer, ThermoFisher Scientific, Waltham, MA, USA). Brains were divided into two halves; one half was fixed in 4% paraformaldehyde for cryosectioning, and the other half was frozen fresh in dry ice before transferring to −80 °C.

### 2.11. Cytokine and Chemokine Arrays

BV2 microglia cells were treated with lipopolysaccharides (LPS) in the presence or absence of calpeptin (CP) for 12–24 h. Cell supernatants were collected, analyzed by Eve Tech’s Discovery cytokine and chemokine arrays (Eve Technologies, Calgary, AB, Canada) for TNF-α, IL-6, monocyte chemoattractant protein-1 (MCP-1), and IP-10. The production of cytokines/chemokines were expressed as pg/mL ± STDEV. Serum samples from vehicle control, MPTP treatment, and CP treated mice were also analyzed by Discovery cytokine and chemokine arrays for determining inflammatory cytokines and chemokines and expressed as pg/mL ± STDEV.

### 2.12. Immunohistochemistry

Cryosections were processed for antigen retrieval in citrate buffer (10 mM Sodium Citrate, 0.05% Tween 20, pH 6.0). Sections were washed in 0.01 M phosphate-buffer saline+0.1% Tritonx-100 (PBST, pH 7.4) and blocked with normal serum for 30 min at room temperature. Sections were incubated in anti-mouse GFAP antibody (Invitrogen, ThermoFisher Scientific, USA, Cat# 14-9892-82) or anti-mouse ROCK2 (Santa Cruz Biotechnology, sc-398519) primary antibody+ 8% normal serum in PBST overnight at 4 °C. Cryosections were washed in PBST. The next day, after overnight incubation in primary antibodies, sections were washed in PBS and incubated in the horse anti-mouse DyLight 488 (DI-2488, Vector Laboratories, Newark, CA, USA) or horse anti-rabbit DyLight 594 secondary antibody (DI-1094, Vector Laboratories) for an hour at room temperature. Sections were washed with PBS and covered with vectashield vibrance antifade mounting medium with DAPI (H-1800, Vector Laboratories).

The images were captured using an Olympus IX73 microscope. The captured images were used to count the cell number using ImageJ software (1.54f, NIH). The ‘Cell Counter’ plugin in ImageJ (Fiji) was used to count the ROCK 2 cells in dorsal striatum sections.

### 2.13. Statistical Analysis

Statistical analyses were conducted using GraphPad Prism (version 6.0) software and Microsoft Excel. Data are presented as mean ± standard deviation (STDEV). Statistical comparisons were performed using a two-tailed paired Student’s *t*-test and one-way ANOVA with the Bonferroni post hoc test to determine the differences between groups. A *p*-value of less than 0.05 was considered statistically significant for all calculations of quantitative data presented here.

## 3. Results

### 3.1. Calpain Activation and Pro-Inflammatory Cytokine Production in Microglia Cells

We began by examining the role of calpain in activation of microglia, the resident immune cells of the central nervous system (CNS). Microglial calpain activation has been implicated in the amplification of neuroinflammatory responses [22]. Microglial responses are regulated by a number of cytokines and chemokines. TNF-α is a cytokine that is known to activate astrocytes and microglia following an insult or injury [31]. Similarly, another pro-inflammatory cytokine IL-6, induces inflammatory response activation [32]. One of the key chemokines, MCP-1, is a member of the C-C chemokine family and is actively involved in inflammation [33]. This chemokine regulates the migration and infiltration of monocytes/macrophages, and its level in serum increases with ischemic stroke and myocardial infarction. Several factors induce its release, such as pro-inflammatory cytokines, oxidative stress, or growth factors [33]. Macrophage inflammatory protein-1α is also a member of the C-C chemokine subfamily [34,35]. LPS induces secretion of chemokines by monocytes, which could attenuate hematopoietic progenitor cell proliferation [36]. Other than peripheral blood cells, several chemokines are secreted by tissues or organs, such as pulmonary vascular smooth muscle cells, bone marrow, epithelial cells, and the brain [34]. In the CNS, it has been shown that IL-1β induced an increase in chemokine secretion by astrocytes [37]. Similarly, LPS, TNF-α, and Il-1β induce secretion of chemokines including MCP-1 and IP-10 by microglial cells [38].

**Figure 2:**
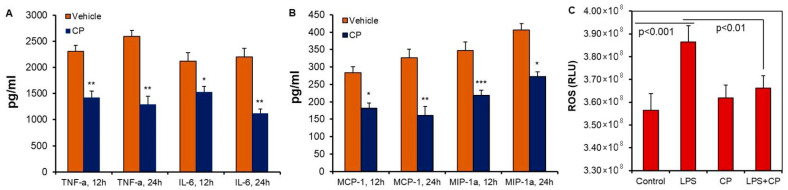
Calpain activation and production of pro-inflammatory cytokines and chemokines by microglial cells. The BV2 murine microglial cell line was grown in collagen-coated T25 flasks. Cells were treated with lipopolysaccharide (LPS) to induce the production of pro-inflammatory cytokines and chemokines. The control group was treated with a vehicle, and the treatment groups received calpeptin (CP), a pan calpain inhibitor. Cell supernatants were collected at 12 h, and 24 h of incubation to monitor the production of several inflammatory cytokines/chemokines. The collected supernatants were analyzed by multiplex assay for (**A**) TNF-α and IL-6, (**B**) MCP-1 and IP-10, and data expressed as pg/mL ± STDEV (* *p* < 0.05, ** *p* < 0.001, *** *p* < 0.01. N = 3). (**C**) Measurement of ROS in the BV2 microglial cell line following overnight treatment with LPS and CP. A significant increase in ROS production following LPS exposure in BV2 cells was detected. Treatment of cells with CP reduced ROS production, suggesting that calpain activation induced ROS production. N = 3.

To investigate the role of calpain in microglial activation and inflammation, BV2 murine microglial cells were treated with lipopolysaccharide (LPS) to induce production of pro-inflammatory cytokines and chemokines (Figure 2). LPS treatment significantly increased the levels of TNF-α, IL-6 (Figure 2A), MCP-1, and IP-10 at 12 and 24 h (Figure 2B) compared to the vehicle-treated control group (* *p* < 0.05, ** *p* < 0.001, *** *p* < 0.01). Calpain inhibition with calpeptin (CP) attenuated the production of these cytokines and chemokines, suggesting that calpain activation is critical for LPS-induced inflammation. Furthermore, LPS treatment increased reactive oxygen species (ROS) production in BV2 cells (Figure 2C). ROS are byproducts of cellular metabolic activity and play a role in regulating cellular homeostasis, though, excessive ROS can contribute to metabolic dysfunction and inflammation. ROS production in BV2 cells was significantly reduced by CP treatment, indicating that calpain activation contributes to ROS generation in microglia (Figure 2C).

### 3.2. Calpain Inhibition Reduces ROS Production and Enhances Cell Survival in Motoneuron Cells

**Figure 3:**
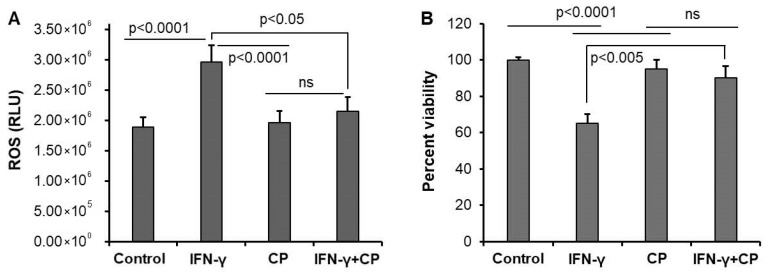
Calpain inhibition decreases ROS production and cell death in VSC4.1 motoneuron cells. (**A**) VSC4.1 cells were treated with either 40 ng/mL IFN-γ or 40 ng/mL IFN-γ + 10 µM CP overnight. ROS assay was performed in 96-well plate by the Reactive Oxygen Species Assay Kit (ab113851) (Abcam, Cambridge, UK). IFN-γ induced production of ROS when the cells were treated overnight. Calpain inhibition reduced ROS production in VSC4.1 cells when the cells were treated with CP along with IFN-γ. Data are representative of three separate experiments. (**B**) VSC4.1 cells treated overnight with IFN-γ (40 ng/mL) or IFN-γ + CP (10 µM) were tested for cell survival by the MTS assay. IFN-γ treatment produced a significant reduction in cell survival, whereas calpain inhibition by CP increased cell viability. ns = no significant difference.

Calpain activation in neurons promotes mitochondrial dysfunction and oxidative stress, creating a perpetuating cycle of cellular damage and death [39]. To explore the potential beneficial effects of calpain-2 inhibition on neuroprotection, we first used an in vitro model: the VSC4.1 cell line. In VSC4.1 motoneurons, IFN-γ treatment induced a significant increase in ROS production (Figure 3A), while the MTS assay showed that IFN-γ treatment reduced cell viability (Figure 3B). Calpain inhibition with CP improved cell survival, and attenuated ROS production, suggesting that calpain activation mediates IFN-γ -induced oxidative stress and cell death in motoneurons. No significant difference was observed between the control and CP-treated groups in the absence of IFN-γ, indicating that CP specifically targets overactivated calpain-mediated pathways.

### 3.3. Calpain Inhibition Reduces Systemic Inflammation in MPTP-Treated Mice

**Figure 4:**
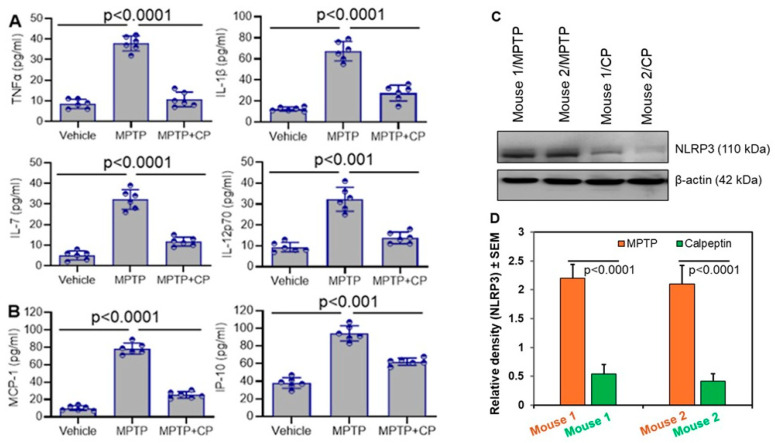
Increased levels of inflammatory cytokines and chemokines are detected in plasma of MPTP mice as compared to vehicle controls. Inhibition of calpain significantly decreased inflammatory cytokines and chemokines compared to MPTP mice. Mice were sacrificed on day 7, and plasma collected from different treatment groups were analyzed for levels of inflammatory cytokines/chemokines. (**A**) Inflammatory cytokines TNF-α, IL-1β, IL-7, and IL-12 were quantitated by Discovery cytokine array. The measurement of cytokine levels in the plasma indicates a significant increase following MPTP exposure compared to the vehicle treatment group. The CP treatment in MPTP mice (MPTP+CP) significantly reduced the level of inflammatory cytokines compared to the levels in the MPTP exposure group. (**B**) Inflammatory chemokines MCP-1 and IP-10 were quantitated by Discovery chemokine array. The MPTP exposure significantly increased the production of these chemokine levels in the plasma. When mice received CP with MPTP exposure (MPTP+CP), the levels of these chemokines were reduced significantly. N = 4–6. (**C**) Western bot analysis showed decreased levels of NLRP3 by CP, suggesting that calpain inhibition may reduce inflammation in MPTP mice. (**D**) Densitometric analysis of protein bands was calculated by ImageJ as described in the methods. Protein quantitation shows significant reduction in NLRP3 protein levels following calpain inhibition in MPTP mice (CP) compared to only MPTP treatment (MPTP). N = 3.

To extend our findings to an in vivo context, we utilized a well-established model of PD: mice treated with MPTP. We began by examining the influence of calpain on neuroinflammation in vivo. Analysis of plasma cytokines and chemokines revealed elevated levels of Tumor necrosis factor-α (TNF-α), IL-1β, IL-7, IL-12, MCP-1, and IP-10 in MPTP-treated mice compared to vehicle controls (Figure 4A,B). TNF-α plays a crucial role in organizing the inflammatory response [31], and its abnormal expression is found in neurodegenerative and inflammatory diseases. The release and production of IL-1β are tightly regulated. Caspase-1 enzyme converts the inactive form of IL-1β into the active form [40]. IL-1β also promotes production of another cytokine, IL-6 [41]. IL6 is a key mediator of neuroinflammation and is released in response to inflammation, injury, or infection. It acts as a signaling molecule that helps the body defend against adverse situations [42]. IL-7 conversely, is a hematopoietic cytokine that plays a critical role in maintaining the balance and stability of the immune system. It does so by regulating the development, proliferation, and survival of B and T cells [43]. IL-12 p70 is a heterodimeric cytokine that is crucial in cell-mediated immunity and Th1 responses [44]. This cytokine promotes the differentiation of naïve T cells into Th1 helper cells, which are essential for priming adaptive immune responses to pathogens recognized by the innate immune system. IL15 is a pro- inflammatory cytokine which favors the development of a strong cellular immune response [45].

Calpain inhibition with CP significantly reduced the levels of these inflammatory mediators, highlighting the systemic anti-inflammatory effects of calpain inhibition in MPTP-induced neurotoxicity (Figure 4A,B). Ongoing neuroinflammation and mitochondrial dysfunction might have induced the NLRP3 protein level in the mouse brain treated with MPTP (Figure 4C). Inhibition of calpain by calpeptin significantly reduced the NLRP3 protein levels in the MPTP-treated mouse brains (Figure 4D). Reduction in neuroinflammation by calpeptin in MPTP-treated mice has played a crucial role in lowering the level of NLRP3. Additionally, elevated levels of NLRP3 are known to stimulate the production of IL-1β, a relationship that is also evident in our data (Figure 4A). The increased level of IL-1β in mice treated with MPTP decreased when mice received calpeptin along with MPTP, suggesting that calpeptin effectively reduced NLRP3 expression.

### 3.4. Calpain Inhibition Attenuates Neuroinflammation and Astrocyte Activation in MPTP-Treated Mice

**Figure 5:**
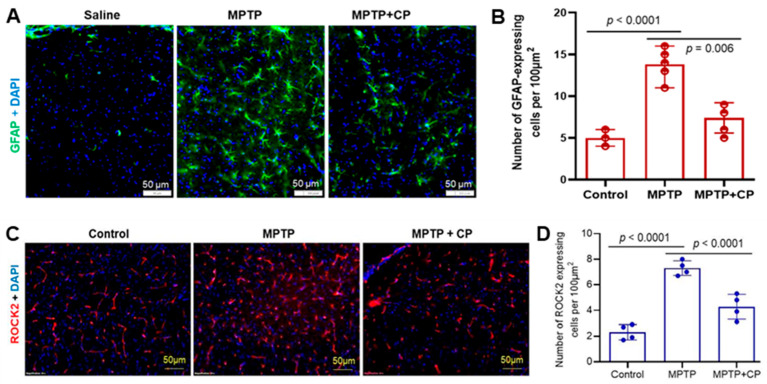
Calpain inhibition reduces MPTP-induced reactive GFAP + astrocytes and ROCK2 expression in vivo in MPTP mice. (**A**) The dorsal striatum in mice from different treatment groups immunostained for astrocyte marker GFAP. GFAP immunostaining of tissue sections indicates that the astrocyte’s number and size increased (activated astrocytes) in the dorsal striatum following sub-acute MPTP exposure (MPTP) in the mice compared to the saline-treated group (Saline). Calpain inhibition by CP in the MPTP exposure group (MPTP+CP), reduced the presence of activated astrocytes. (**B**) Quantitation of the GFAP-positive astrocytes using ImageJ showed that the MPTP exposure significantly (*p* < 0.005) increased the GFAP-positive astrocyte number (astrogliosis), indicating neuroinflammation in the brain area involved in the pathophysiology of PD. The number of active astrocytes was significantly reduced (*p* < 0.001) by calpain inhibition, supporting the role of calpain activation following MPTP exposure in neuroinflammation. N = 3–5. (**C**) ROCK2 immunostaining indicates that the ROCK2-positive cell numbers increased in the dorsal striatum following MPTP treatment compared to control and MPTP+CP groups. Calpain inhibition by CP in MPTP exposed mice reduced the density of ROCK2-positive cells. (**D**) The quantitation of the ROCK-2 positive cells using ImageJ is presented in the bar graph. Statistical analysis showed a significant increase in ROCK-2 positive cells in the dorsal striatum following MPTP exposure in the mice. CP treatment significantly reduced the number of cells expressing ROCK2 in MPTP + CP treatment group compared to the MPTP exposure group. N = 4.

We next examined the influence of calpain on astrocytes in vivo. These glial cells are essential components of blood–brain barrier (BBB) and maintain neuronal homeostasis. Phenotypic changes are evident in astrocytes following neuronal insults along with molecular changes [46,47]. The number of activated astrocytes increases during neuroinflammation at the affected site. These activated astrocytes can be easily identified due to their distinct phenotype, such as an enlarged cell body and elongated, branched processes. We used GFAP immunostaining to observe such changes in the dorsal striatum following exposure to MPTP (Figure 5A). Further, calpain inhibition with CP reduced the number and size of activated astrocytes (Figure 5A, MPTP+CP)**.** Quantitation of GFAP-positive astrocytes revealed a significant increase following MPTP exposure compared to saline and MPTP+CP groups (Figure 5B). The significant reduction in GFAP-positive astrocytes suggests that MPTP-induced neuroinflammation is attenuated by CP treatment (Figure 5A,B).

Calpain-2 has been shown to cleave ROCK2 (Rho-associated coiled-coil containing protein kinase 2), a regulator of actin cytoskeleton dynamics [48]. We found that MPTP exposure increased the number of ROCK2-positive astrocytes in the dorsal striatum, while CP treatment significantly reduced ROCK2-positive cell numbers (Figure 5C,D). These results, along with those shown in Figure 4, indicate that calpain activation contributes to neuroinflammation and neurodegeneration in MPTP-induced PD pathology.

### 3.5. Calpain-2 Knockdown Reduces Antigen Presentation and Production of Inflammatory Cytokines and ROS in Human Microglia

In addition to being involved in immune activation and neuroinflammation, microglia also can present antigens to CD4+ T cells [49]. In a mouse model of PD, there is an increase in a subset of CD4+ T cells that may be involved in neuroinflammation [8,50]. Calpain inhibition reduces the presence of these CD4+ T cells, suggesting that calpain activation may enhance the activity of this subtype of CD4+ T cells and may contribute to the disease process.

**Figure 6:**
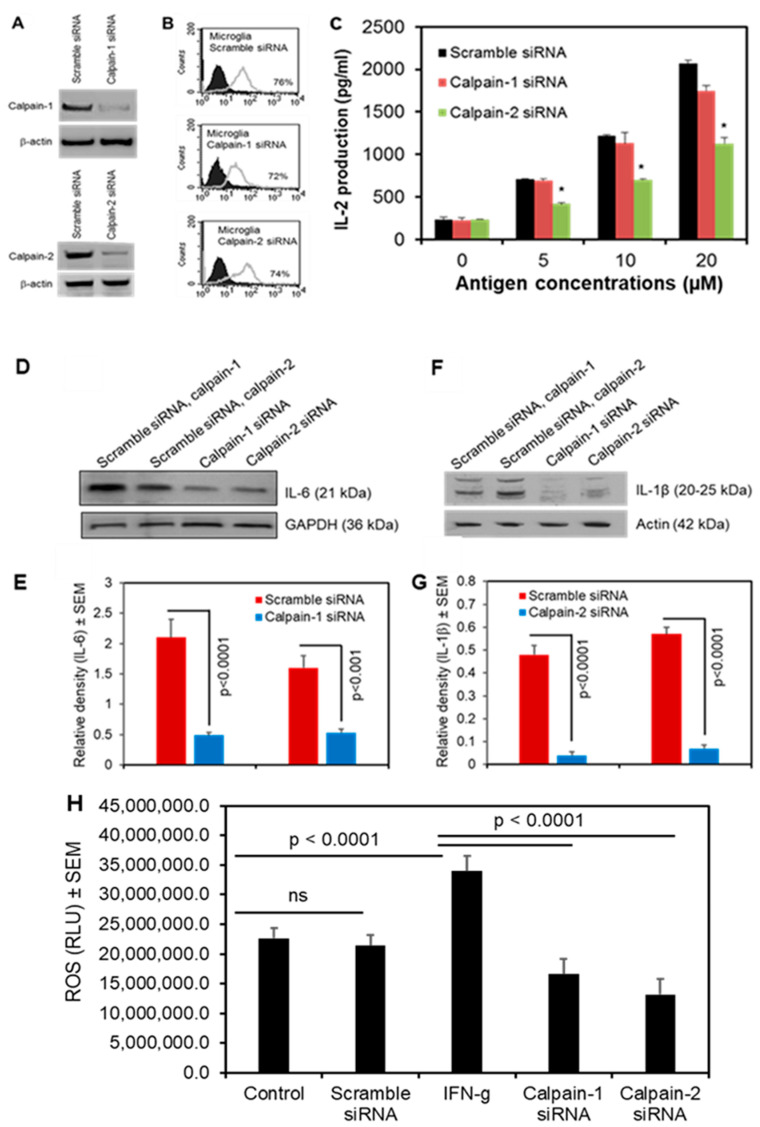
Inhibition of calpain-2 by siRNA-mediated knockdown assay decreases antigen presentation to CD4^+^ T cells and reduces inflammatory cytokines and ROS production in microglia. Human microglia-SV40 cell line was transduced with HLA-DR4 by retroviral gene transfer assay. Cells were then subjected to *calpain-1* and *calpain-2* knockdown as described in the methods. (**A**) Western Blot analysis confirmed knockdown of calpain-1 and calpain-2 proteins in human microglia-SV40 cells. (**B**) Flow cytometric analysis of microglia-SV40 for HLA-DR4 after siRNA-mediated knockdown of calpain-1/2. (**C**) *Calpain-1* and *calpain-2* knockdown cells were incubated with IgG kappa antigen overnight, washed and co-cultured with the IgG k188-203 peptide specific T cell hybridoma (2.18a) cells for 24 h. The production of IL-2 in the culture supernatant was measured by ELISA as an indicator of T cell response. Data presented in the bar graph suggests that the knockdown of *calpain-2* (calpain-2 siRNA) significantly inhibited activation of CD4+ T cells compared to control (scrambled siRNA) and knockdown of *calpain-1* (calpain-1 siRNA). * *p* < 0.005. (**D**–**G**) Knockdown of *Calpain-1*, and *Calpain-2* Reduces Pro-inflammatory Cytokines. Western blot (WB) analysis demonstrated a significant reduction in IL-6 (**D**) and IL-1β (**F**) protein expression levels following the silencing of *calpain-1* and *calpain-2*. Quantification of WB data shows that siRNA knockdown of *calpain-1* and *calpain-2* in SV-40 cells resulted in a significant decrease in the protein levels of the pro-inflammatory cytokines IL-6 (**D**,**E**), *p* < 0.0001 and *p* < 0.001, respectively); and IL-1β (**F**,**G**), both *p* < 0.0001) compared to the control scramble siRNA. (**H**) ROS assay suggests that inhibition of *calpain-1* and *calpain-2* by siRNA significantly inhibited (*p* < 0.0001) IFN-γ-induced ROS production compared to SV40 microglia cells stimulated with IFN-γ.

We next investigated the roles of specific calpain isoforms in antigen presentation. To explore the role of calpain in microglial antigen presentation, human microglia-SV40 cells were subjected to siRNA-mediated knockdown of *calpain-1* and *calpain-2* (Figure 6A). These cells were transduced with HLA-DR4, which is crucial for antigen presentation via the MHC class II pathway. Flow cytometric analysis confirmed the expression of HLA-DR4 in these cells (Figure 6B). Interleukin-2 (IL-2) is a pleiotropic cytokine primarily produced by activated CD4+ T cells. This cytokine plays a crucial role in T cell growth, proliferation, and differentiation. Additionally, IL-2 production occurs rapidly—a short-lived event following the recognition of an antigen [51]. Knockdown of *calpain-2* (but not *calpain-1*) significantly reduced IL-2 production by CD4+ T cells in response to antigen presentation (Figure 6C), indicating that calpain-2 plays a specific role in microglial antigen presentation and T cell activation.

We also investigated the levels of pro-inflammatory cytokines IL-6 and IL-1β after knocking down *calpain-1* and *calpain-2* using siRNA in microglia cells. Quantification of protein levels, assessed by Western blot analysis showed a significant inhibition in IL-6 (Figure 6D) and IL-1β (Figure 6F) following the silencing of both *calpain-1* and *calpain-2* in microglial cells. Furthermore, the inhibition of *calpain-1* and *calpain-2* by siRNA significantly inhibited ROS production in these microglial cells when stimulated with IFN-γ (Figure 6H).

**Figure 7:**
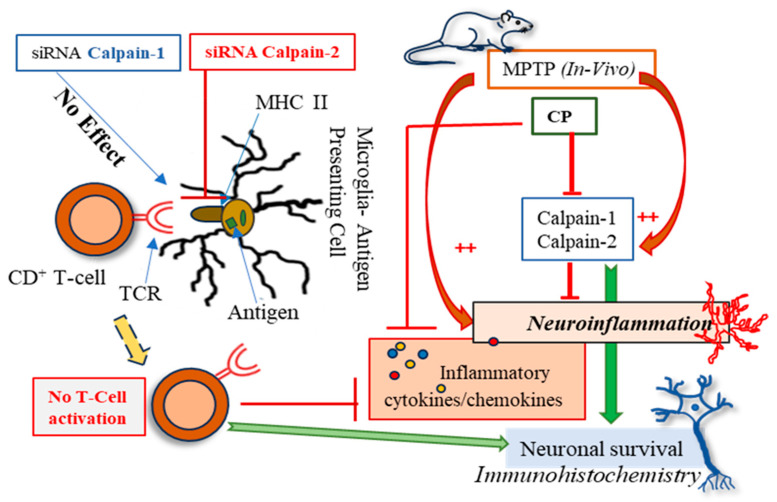
This sketch diagram shows that in both the in vivo (MPTP) and in vitro *(siRNA calpain-1/calpain-2)* models, the toxin induced an increase in calpain11 and calpain-2 expression along with neuroinflammation, evident by the presence of an increased number of activated astrocytes in the dorsal striatum and dysregulation of cytokines/chemokines in the rat plasma. Intervention with a pan calpain inhibitor (calpeptin) reduced neuroinflammation and levels of inflammatory cytokines and chemokines, which may prevent neurodegenerative changes. Inhibition of *calpain-2* (siRNA) reduced antigen presentation to CD4+ T cells, which may have decreased the release of inflammatory cytokines and chemokines, thereby preventing neuronal damage.

These findings demonstrate that calpain activation plays a critical role in neuroinflammation, oxidative stress, and neurodegeneration in both in vitro and in vivo models as summarized in Figure 7. Calpain inhibition attenuated pro-inflammatory cytokine production, reduced ROS generation, and protected against MPTP-induced neurotoxicity. Thus, targeting calpain activity may be a promising therapeutic strategy for neurodegenerative diseases such as PD.

## 4. Discussion

Calpain plays a critical role in PD by contributing to the degeneration of dopaminergic neurons in the substantia nigra [8]. Its overactivation, triggered by calcium overload, disrupts cellular homeostasis, and leads to mitochondrial dysfunction, increasing oxidative stress and reducing energy production [16]. Additionally, calpain activation in glial cells drives neuroinflammation through the release of pro-inflammatory cytokines and induction of neuronal damage by inducing apoptosis via caspase activation and cytoskeletal protein cleavage. While there are many calpain isoforms, calpain-1 and calpain-2 play critical roles in cellular processes including glial activation, signal transduction, cytoskeletal remodeling, and cell survival/death [17,52]. However, their dysregulation has been implicated in both inflammation and neurodegeneration, contributing to the pathogenesis of various neurological disorders, including PD, AD, and ALS [8,53,54,55,56,57]. The present study demonstrates the critical role of calpain activation in mediating neuroinflammation, oxidative stress, and neurodegeneration in both in vitro and in vivo models. Our findings highlight that calpain inhibition attenuates pro-inflammatory cytokine production, reduces ROS generation, and protects against neurotoxicity, suggesting that calpain may be a key participant in the pathophysiology of neurodegenerative diseases such as PD. In microglial cells, LPS-induced activation led to a significant increase in pro-inflammatory cytokines (TNF-α, IL-1β) and chemokines (IP-10, MCP-1), which were attenuated by calpain inhibition. This aligns with previous studies showing that calpain activation is involved in microglial activation and the subsequent release of inflammatory mediators [22]. Additionally, LPS-induced ROS production was significantly reduced by calpain inhibition, suggesting that calpain activation contributes to oxidative stress in microglia. These findings are consistent with the known role of calpain in regulating NADPH oxidase activity, a major source of ROS in immune cells [16]. Together, these results underscore the importance of calpain in mediating neuroinflammation and oxidative stress, both of which are hallmarks of neurodegenerative diseases.

Pro-inflammatory chemokines, MCP-1 and IP-10 levels increased in the plasma of mice following MPTP exposure. However, calpeptin treatment reduced this increase in chemokine levels. The chemokine MCP-1 has been found to play an active role in various diseases, particularly neurodegenerative conditions such as AD, PD, and multiple sclerosis [58]. Additionally, MCP-1 is critically involved in insulin resistance (elevated levels of this chemokine in circulation are indicative of both type 1 and type 2 diabetes). Another chemokine, Interferon Gamma-induced Protein 10 (IP-10), appears to be a very promising biomarker for the detection of “cytokine storm” following an immune response in inflammatory diseases [59]. As its name suggests, it is secreted in response to IFN-γ. Therefore, the increased plasma cytokine level following MPTP exposure suggests commensurately increased IFN-γ. However, calpeptin reduced the level of this cytokine, indicating that calpain inhibition must have reduced the level of IFN-γ as well.

Some inflammatory chemokines are also critical to immune responses and inflammation as observed with elevated levels in a mouse model of acute respiratory distress syndrome [60]. The authors also observed that the presence of IL-1α and TNF-α was required to increase its release [61]. IP-10 is also a member of the CC chemokine family and plays a crucial role in immune cell activation. The presence of TNFα, IL-1β, and IFN-γ induces its release from CNS endothelial cells, vascular smooth muscle cells, and microglial cells. Additionally, IL-7 induces monocyte production [35]. Reactive astrocytes in patients with AD show upregulation of IP-10 and other chemokines [58]. Recently, a longitudinal study in PD patients showed elevated levels of chemokines in cerebrospinal fluid (CSF), which correlates with cognitive impairment in these patients. [62]. The MPTP toxin treatment in mice shows elevated levels of these cytokines (TNFα, IL-1β, and IL-7) in the plasma, suggesting that they might have contributed to the induction and release of inflammatory factors in the MPTP-exposed mice. Since calpain also modulates inflammatory mediators, its inhibition by calpeptin significantly reduces the inflammatory response in the MPTP+CP treatment group.

In the MPTP mouse model of PD, calpain inhibition reduces astrocyte activation and ROCK2 expression in the dorsal striatum. Our previous research also indicated that inhibiting calpain decreases neuroinflammation in the nigrostriatal pathway in animal models of PD [8]. Additionally, it has been shown that suppressing ROCK2 activation reduces pro-inflammatory cytokines and alleviates disease severity in patients with irritable bowel disease [63]. This suggests that calpain inhibition leads to reduced ROCK2 activation and consequently, decreased neuroinflammation. These findings are consistent with previous studies showing that calpain activation contributes to astrogliosis and neurodegeneration in PD models [8,64,65]. The reduction in GFAP-positive astrocytes and ROCK2-positive cells following calpain inhibition suggests that calpain activation drives neuroinflammation and neuronal damage in PD. Furthermore, the systemic reduction in inflammatory cytokines and chemokines in MPTP-treated mice following calpain inhibition highlights the broad anti-inflammatory effects of calpain inhibition. Calpain inhibitors such as calpeptin (pan calpain inhibitor) and calpain-2 siRNA demonstrated significant neuroprotective effects in our models, supporting their potential for clinical development. MPTP-treated mice showed elevated pro-inflammatory cytokines (TNFα, IL-1β, IL-7, IL-12) and chemokines (MCP-1, IP-10), indicating neuroinflammation. TNFα promotes TNFR1-mediated cell death [66,67], while IL-1β contributes to CNS pathology [68]. Calpeptin reduced the increased level of these cytokines, suggesting calpain inhibition mitigates inflammation. Increased IL-7 and IL-12 reflect immune dysregulation [44,46], while MCP-1 elevation supports its potential as a PD biomarker [43]. Moreover, IL-1β modulates both innate and adaptive immune cells [45]. MPTP exposure elevated TNF-α level, which was reduced by calpain inhibition, reinforcing the relationship between calpain activity and pro-inflammatory cytokine production. Notably, a significant increase in plasma MCP-1 levels following exposure to MPTP suggests the presence of neuroinflammation-induced reactive astrocytes and microglia in the brain, which may lead to an increased release of this chemokine. IP-10 upregulation suggests IFN-γ involvement, which calpeptin counteracts by reducing ROS and chemokine levels. Inflammatory chemokines induced by TNFα, IL-1β, and IL-7 [33,35], also decreases with calpain inhibition. Furthermore, the significantly reduced NLRP-3 protein level in the MPTP+calpeptin treatment group, compared to MPTP-treated mice, supports calpeptin’s protective role in reducing neuroinflammation. These findings demonstrate calpain’s role in neuroinflammation and highlight calpain inhibition as a potential therapeutic strategy for PD-like pathology.

The heightened presence of CD4+/CD8+ T cells, microglia, and astroglia has been observed in the substantia nigra of PD patients and MPTP-induced PD models [69,70,71]. These circulating T cells might have been activated by alpha-synuclein (α-syn), the key pathological protein involved in the pathogenesis of PD [72]. Microglia play a crucial role in the processing and presentation of these antigens to CD4+ T cells. The activation of calpain-2 may be responsible for triggering this neuroinflammatory response by activating microglia [50,70]. Further, we found increased expression of a subset of CD4+ T cells in MPTP-induced mouse model of PD, which was diminished by calpain inhibition [69]. These findings strongly suggest that calpain activation is related to CD4+ T cell activation response. To distinctly identify this isoform specific immune response, we used siRNA-mediated knockdown of *calpain-1* and *calpain-2*. Our siRNA-mediated knockdown experiments revealed that calpain-2, but not calpain-1, plays a specific role in antigen presentation by human microglia. Knockdown of *calpain-2* significantly reduced IL-2 production by CD4+ T cells, indicating that calpain-2 regulates microglial antigen presentation and T cell activation. This novel finding suggests that calpain-2 may play a role in the adaptive immune response in neurodegenerative diseases [73]. Further studies are needed to explore the mechanisms by which calpain-2 regulates antigen presentation and its implications for neuroinflammation. Interestingly, the ROS data and pro-inflammatory cytokine levels of IL-6 and IL-1β following silencing of *calpain-1* and *calpain-2* suggest that both isoforms of calpain play active roles in ROS production and the onset of inflammation in the cellular system.

Targeting calpain activity has emerged as a potential therapeutic strategy for neurodegenerative diseases. Calpain inhibitors have shown promise in preclinical studies by reducing neuroinflammation, preventing protein aggregation, and protecting neurons from degeneration [74,75]. However, the challenge lies in selectively modulating calpain-1 and calpain-2 activities without disrupting their physiological functions. The balance between calpain-1 and calpain-2 activities is also crucial for maintaining cellular homeostasis. While calpain-1 may initially exert protective effects, its overactivation, along with calpain-2, can lead to a vicious cycle of inflammation and neurodegeneration. For instance, in PD models, calpain activation has been shown to contribute to the neuroinflammatory response and dopaminergic neuron loss, highlighting its dual role in disease progression [8]. In the mammalian brain, calpain-1 and calpain-2 isoforms are ubiquitously expressed. Activation of calpain-2 is detected following neuronal injury and neuroinflammation [8,50,76], suggesting that inhibition of calpain-2 might prevent neuronal injury. In VSC4.1 motoneuron cells, IFN-γ-induced ROS production and cell death were significantly reduced by calpain inhibition. The protective effects of calpain inhibition on cell viability further support the potential therapeutic value of targeting calpain in neurodegenerative conditions characterized by oxidative stress and neuronal loss, such as ALS and PD [77]. The observed neuroprotective effects of calpain-2 inhibition highlight its potential as a therapeutic target for promoting neuronal repair and regeneration in neurodegenerative diseases.

## 5. Limitations

While our study provides compelling evidence for the role of calpain in neuroinflammation and neurodegeneration, there are some limitations. First, the in vitro models used in this study do not fully represent the complexity of neurodegenerative diseases in humans. Second, the MPTP mouse model, while widely used, does not manifest the progressive nature of PD. Future studies using additional animal models and human samples will be necessary to validate these findings. The in vitro models used in this study, such as the BV2 microglial cell line and VSC4.1 motoneuron cells, provide a controlled environment to investigate specific mechanisms. However, these models may not fully replicate the complexity of neurodegenerative diseases in humans.

Immortalized cell lines like BV2 and VSC4.1 may not adequately represent the behavior of primary microglia or neurons, which exhibit more diverse and dynamic responses in vivo. In vitro models lack the intricate interactions between neurons, glia, and immune cells that occur in the brain. These interactions are critical for understanding the full scope of neuroinflammation and neurodegeneration. The use of LPS and IFN-γ as inflammatory or neurotoxic stimuli also provides a simplified representation of the multifactorial triggers involved in human neurodegenerative diseases. The exact mechanisms by which calpain-2 regulates antigen presentation remain unclear. Further studies are needed to explore whether calpain-2 affects MHC-II expression, antigen processing, or co-stimulatory molecule expression. While this study provides important insights into the role of calpain in neurodegeneration, its limitations highlight the need for further research to fully understand the therapeutic potential of calpain inhibition. Addressing these limitations will be critical for developing safe and effective calpain-based therapies for neurodegenerative diseases.

## 6. Conclusions

The findings of this study underscore the pivotal role of calpain activation in neuroinflammation, oxidative stress, and neurodegeneration, providing compelling evidence for its involvement in the pathophysiology of neurodegenerative diseases such as PD. By employing both in vitro and in vivo approaches, we demonstrated that calpain inhibition attenuates pro-inflammatory cytokine production, reduces ROS generation, and protects against neurotoxicity. These results highlight the therapeutic potential of targeting calpain in neurodegenerative conditions characterized by chronic inflammation and neuronal loss. The consistent neuroprotective effects of calpain inhibition across multiple models suggest that calpain inhibitors could be promising candidates for the treatment of neurodegenerative diseases. By targeting calpain, it may be possible to simultaneously address multiple pathological processes, including neuroinflammation, oxidative stress, and neuronal loss. However, further studies are needed to evaluate the safety and efficacy of calpain inhibitors in human trials, as well as to explore the specific roles of calpain-1 and calpain-2 in different neurodegenerative conditions. In conclusion, this study demonstrates that calpain activation is a central driver of neuroinflammation, oxidative stress, and neurodegeneration. Calpain inhibition attenuates these pathological processes and promotes neuronal survival and differentiation, highlighting its potential as a therapeutic target for neurodegenerative diseases.

## Data Availability

The data used to support the findings of this manuscript are available from the corresponding authors upon reasonable written request after the publication.
